# Real-world experience with ultrasound renal denervation utilizing home blood pressure monitoring: the Global Paradise System registry study design

**DOI:** 10.1007/s00392-023-02325-x

**Published:** 2023-11-09

**Authors:** Felix Mahfoud, Michel Azizi, Joost Daemen, Andrew S. P. Sharp, Atul Patak, Juan F. Iglesias, Ajay Kirtane, Naomi D. L. Fisher, Andrea Scicli, Melvin D. Lobo

**Affiliations:** 1 Klinik für Innere Medizin III, Saarland University Hospital, Homburg/Saar, Germany; 2 Institute for Medical Engineering and Science, Massachusetts Institute of Technology, Cambridge, MA USA; 3 Université Paris Cité, 75006 Paris, France; 4 AP-HP, Hôpital Européen Georges-Pompidou, Hypertension Department and DMU CARTE, 75015 Paris, France; 5 INSERM, CIC1418, 75015 Paris, France; 6 Department of Cardiology, Erasmus MC, University Medical Center Rotterdam, Rotterdam, The Netherlands; 7 University Hospital of Wales, Cardiff and Cardiff University, Cardiff, UK; 8 Department of Cardiovascular Medicine, Princess Grace Hospital, Monaco and University of Toulouse, Toulouse, France; 9 Department of Cardiology, Geneva University Hospitals, Geneva, Switzerland; 10 Columbia University Medical Center/New York-Presbyterian Hospital and the Cardiovascular Research Foundation, New York, NY USA; 11 Division of Endocrinology, Diabetes and Hypertension, The Brigham and Women’s Hospital, Boston, MA USA; 12 Recor Medical, Inc., Palo Alto, CA USA; 13 Barts NIHR Biomedical Research Centre, William Harvey Research Institute, Queen Mary University of London, London, UK

**Keywords:** Hypertension, Renal denervation, Ultrasound, Home blood pressure, Registry, Patient-reported outcomes

## Abstract

**Background:**

Hypertension is a major public health issue due to its association with cardiovascular disease risk. Despite the availability of effective antihypertensive drugs, rates of blood pressure (BP) control remain suboptimal. Renal denervation (RDN) has emerged as an effective non-pharmacological, device-based treatment option for patients with hypertension. The multicenter, single-arm, observational Global Paradise™ System (GPS) registry has been designed to examine the long-term safety and effectiveness of ultrasound RDN (uRDN) with the Paradise System in a large population of patients with hypertension.

**Methods:**

The study aims to enroll up to 3000 patients undergoing uRDN in routine clinical practice. Patients will be recruited over a 4-year period and followed for 5 years (at 3, 6, and 12 months after the uRDN procedure and annually thereafter). Standardized home BP measurements will be taken every 3 months with automatic upload to the cloud. Office and ambulatory BP and adverse events will be collected as per routine clinical practice. Quality-of-Life questionnaires will be used to capture patient-reported outcomes.

**Conclusions:**

This observational registry will provide real-world information on the safety and effectiveness of uRDN in a large population of patients treated during routine clinical practice, and also allow for a better understanding of responses in prespecified subgroups. The focus on home BP in this registry is expected to improve completeness of long-term follow-up and provide unique insights into BP over time.

**Graphical abstract:**

Global Paradise System registry study design. ABP, ambulatory blood pressure; BP, blood pressure; FU, follow-up; M, month; OBP, office blood pressure.

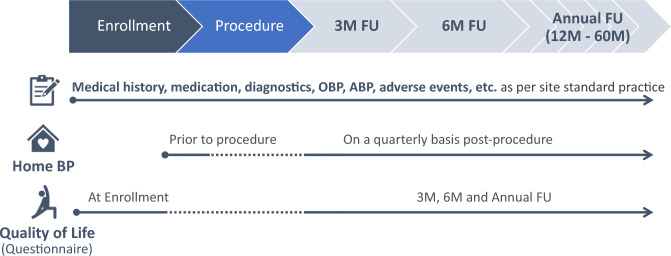

## Introduction

Hypertension is highly prevalent [1, 2] and is the most common modifiable risk factor for cardiovascular disease [3]. A number of factors contribute to uncontrolled hypertension, including inappropriate treatment (doses and combinations) due to physician inertia and poor medication adherence [4, 5]. In addition, a subset of patients have hypertension that is truly resistant to optimal guideline-directed triple pharmacotherapy (in whom adherence has been confirmed) [6–9].

Renal denervation (RDN) has emerged as an effective non-pharmacological treatment option for patients with uncontrolled and resistant hypertension [10, 11]. Ultrasound RDN (uRDN) with the Paradise™ Ultrasound Renal Denervation system (Recor Medical, Palo Alto, CA, USA) has been shown to be safe and effective, resulting in significant and durable reductions in BP in the presence and absence of antihypertensive drugs [12–18].

As the number of patients treated with uRDN in clinical practice increases, the GPS Registry has been set up to document the real-world long-term safety and effectiveness of uRDN in patients treated in routine clinical practice.

## Methods

### Study design

**Fig. 1:**
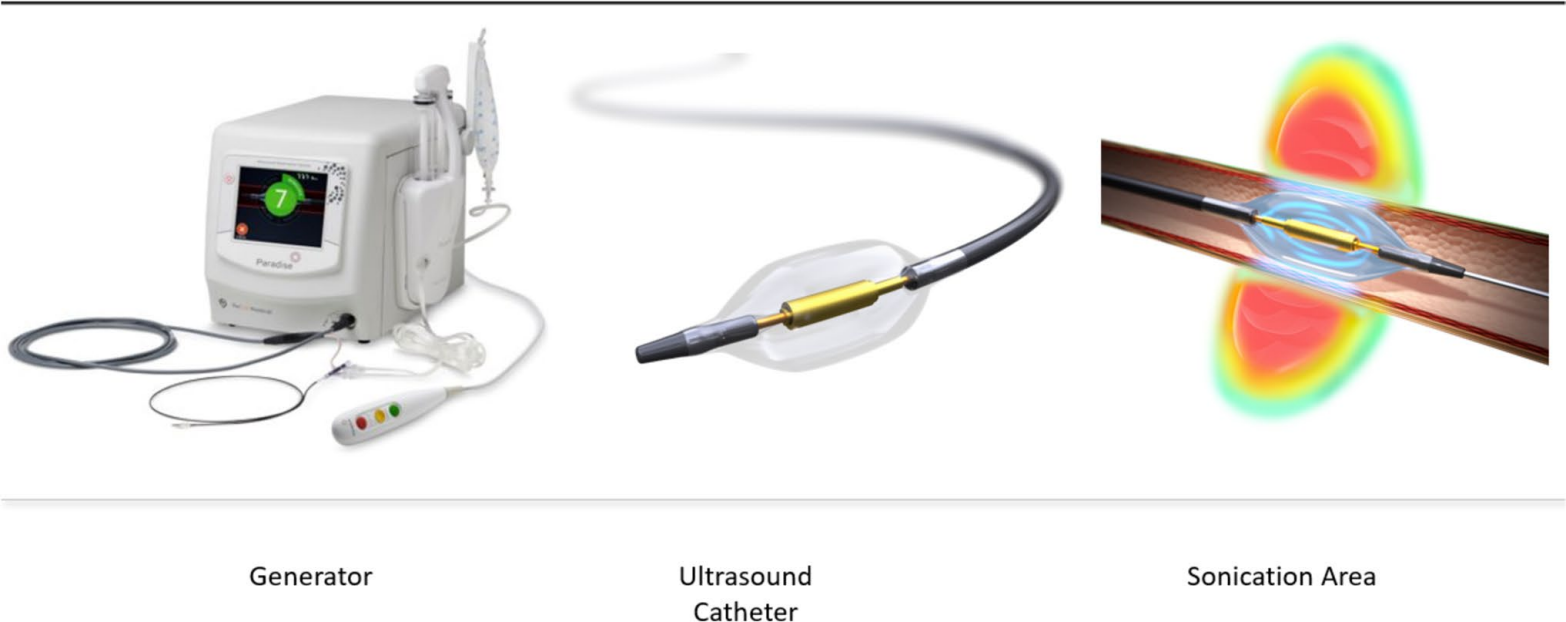
Paradise ultrasound renal denervation system

The GPS Registry (NCT05027685) is a multicenter, single-arm, observational study (Fig. 1). Up to 200 study centers from areas where the Paradise System (Recor Medical, Palo Alto, USA) is available will enroll patients, including but not limited to the European Union, the United Kingdom, Switzerland and Monaco. Additional locations may be added to the GPS Registry over time. Monitoring of study sites will be performed during the registry to assess continued adherence to the protocol and applicable regulations. The recommended follow-up is 5 years.

All national and local approvals will be obtained prior to beginning the study. Any additional requirements imposed by the institutional review board/ethics committee (IRB/EC) or regulatory authority will be followed where appropriate. Annual IRB/EC renewals will be obtained throughout the duration of the registry as per local/country requirements. Written informed consent will be obtained from all patients or their legally designated representative, as defined by local regulations, in accordance with the Declaration of Helsinki.

The GPS Registry will be run under the guidance of a Steering Committee that will include international physicians with expertise in the areas of RDN, vascular medicine, hypertension, cardiology, interventional cardiology, radiology, and nephrology.

### Study population

Based on the international guidelines that have been recently published, patients qualifying for renal denervation should have been unsuccessful in controlling their blood pressure with lifestyle changes and antihypertensive medications. Patients selected for uRDN with the Paradise System as per routine clinical practice on patient selection for renal denervation can be enrolled in the registry. The goal of the study is to collect real-world data on current clinical practices at each participating site. Full details of the study inclusion and exclusion criteria are shown in Table 1. For prospectively enrolled patients, the uRDN procedure is to be scheduled within 30 days of registry enrollment, while retrospectively enrolled patients need to have undergone uRDN within the previous 6 months.

**Table 1:** Inclusion and exclusion criteria

Inclusion criteria
Age ≥ 18 years at time of consent
Candidate for uRDN with the Paradise System based on physician’s assessment
OR
Treated with uRDN using the Paradise System within the 6 months prior to consent
Willing to provide written informed consent (appropriately signed and dated) and agreed to have all study procedures performed

Exclusion criteria
Stented renal artery
Age < 18 years
Pregnancy
Known allergy to contrast medium not amenable to treatment
Renal arteries with diameter < 3 mm and > 8 mm^a^
Renal artery with fibromuscular disease^a^
Renal artery aneurysm or dissection^a^
Renal artery stenosis of any origin > 30%^a^
Iliac/femoral artery stenosis precluding insertion of the Paradise catheter^a^

^a^Determined at time of procedure

### Renal denervation procedure

The Recor Paradise System is a catheter-based system that delivers ultrasound energy to thermally ablate and disrupt the renal efferent and afferent sympathetic nerves, with the goal of achieving a reduction in systemic arterial BP and mitigating end-organ effects due to sympathetic over-activity. The system includes a single-use 6F catheter and an automated, portable, customized generator. The Paradise Catheter is intended to be employed in a catheterization laboratory under fluoroscopic guidance via femoral access only. The catheter consists of a through-lumen shaft with a cylindrical piezoelectric ceramic transducer located at the distal end of the catheter. The catheter has a distal balloon, which is pressurized by the generator to 1.5–2.0 atm using sterile circulating water.

The device's ultrasound transducer converts electrical energy into acoustic energy, which is then delivered radially through the cooling balloon into the renal artery. The pressurized balloon centers the ultrasound transducer within the artery, and the circulation of fluid serves as coolant to protect the endothelial and medial layers of the renal arterial wall. Each catheter has an embedded chip that communicates directly with the generator and specifies the power settings to be applied.

The uRDN procedure will be performed according to routine practice at each study site and consistent with the device’s instructions for use. As treatment is bilateral, specific angiographic requirements need to be met to deem anatomic eligibility. Measurements of the distal, mid, and proximal renal artery diameters are used to select the appropriate Paradise catheter balloon size. The treatment strategy requires operators to deliver a minimum of two (to three) sonications in each main renal artery and the first sonication should be delivered at a distance of at least 5 mm from the artery bifurcation. The Instructions for Use includes details for balloon sizing and treatment strategy regarding the number of emissions per artery and also includes instructions for additional sonications in case there are proximal bifurcations or accessory arteries. Physicians new to the technology will receive proctoring support for initial cases as needed.

Procedural support by a sponsor representative is available to all sites regardless of experience with the Paradise Renal Denervation System.

### Assessments and data collection

Follow-up visits are scheduled at 3, 6, and 12 months after the uRDN procedure and every 12 months thereafter. Each visit will include medical review, measurement of office BP, medication name and dose). Ambulatory BP data (mean values, heart rate, standard deviation) obtained during routine clinical practice at study centers (including daytime, nighttime and 24-h BP) will be recorded and assigned to the nearest follow-up visit.

Other data collected during routine clinical practice will also be reviewed as part of patient follow-up, including any of the following: physical parameters including body weight, HR, seated and standing office BP; smoking status during follow-up, socioeconomic, and healthcare resource use parameters; electrocardiogram; non-invasive cardiac imaging; urinalysis including micro/macroalbuminuria; blood chemistry (serum creatinine, electrolytes, blood glucose and HbA1c, blood lipids); non-invasive renal artery imaging; any other cardiovascular procedures and treatments including glucose- and cholesterol-lowering drugs and antiplatelet/anticoagulant use, and the occurrence of any cardiovascular/cerebrovascular event (MI, HF, AF, etc.) and cardiovascular or non-cardiovascular death.

### Home blood pressure

All clinical sites will be provided with validated, commercially available, validated telemetric home BP monitoring (HBPM) devices BPM Connect Pro, WITHING, Issy-les-Moulineaux, France) [19]. The collection of home blood pressure with this WITHING device is specific to the execution of the study and is not currently part of the sites' routine clinical practice. Prospectively enrolled subjects who elect to be part of HBPM data collection will be provided with a device and will be instructed to record their home BP according to the American Heart Association and American Medical Assocation [20] for seven consecutive days prior to the uRDN procedure. Over this period, subjects will take two measurements in the morning and two measurements in the evening. Consistent with current recommendations [20, 21], patients will be instructed to avoid smoking, caffeinated beverages, or exercise in the 30 min prior to home BP measurement, to take readings in a quiet room after sitting quietly for 5 min, to sit with their back straight and supported and feet flat on the floor with the arm supported on a flat surface and the upper arm at heart level. The same arm with the highest BP determined previously will be used for all home BP measurements, and at least two measurements should be taken 1–2 min apart before taking medications in the morning, and before taking medications and before dinner in the evening. All home BP measurements will be transmitted automatically via cellular or wifi signal by the HBPM device to a dedicated secured cloud-based platform (Study Pal; DELVE; www.delvehealth.com), which is GDPR 2016/679 compliant. The use of this integrated Global System for Mobile communication functionality removes the need and burden for participants to record home BP measurements in a diary.

Subjects will be reminded by automatic text message to measure their home BP if their home BP assessment is incomplete. Ideally seven- but a minimum of 3-day HBPM data (at least twelve valid measurements) are recommended prior to the procedure. Patients participating in home BP data collection will measure their home BP every 3 months during follow-up, ideally over 7 days of measurements with four readings per day prior each visit at the study site. If insufficient baseline home BP data are collected, no additional home BP data will be collected for that patient. Subjects who are unwilling and/or unable to comply with HBPM requirements will not take part in HBPM data collection but will continue to be part of the registry. Retrospectively enrolled subjects will not be provided with a HBPM device, and any available BP data (office, HBP, ABPM) from prior to the uRDN procedure will be collected retrospectively.

### Patient-reported outcomes

The validated Short Form (SF)-12 questionnaire is a 12-item questionnaire that gathers data on patient functional health and well-being. The patient may elect to complete the SF-12 quality-of-life questionnaire as part of the study specific assessments. If patients agree to complete the questionnaire, they will be asked to complete the questionnaire in their local language at enrollment, at 3, 6, and 12 months after the uRDN procedure, and annually thereafter.

### Safety

All centers participating in the GPS Registry will use the Paradise uRDN system per their normal practice. Therefore, any device-related adverse events will be reported to the appropriate regulatory authorities through the Recor Medical vigilance reporting process. Underlying diseases/pre-existing conditions will not be reported as adverse events unless there has been a substantial increase in the severity or frequency of the problem that cannot be attributed to natural history during the course of the registry. The assessment of safety includes but is not limited to collecting the following events: incidence of new onset end-stage renal disease (eGFR < 15 mL/min/m^2^ or need for renal replacement therapy), significant decline in renal function, new renal artery stenosis > 70% by CTA/MRA, incidence of renal artery perforation or dissection requiring intervention, incidence of hospitalizations for hypertensive crisis or symptomatic hypotension.

### Data collection and storage

Subject data will be collected into an electronic data capture system. The data will be monitored and audited for data collection to assess continued compliance with the protocol and applicable regulations to ensure the integrity of the data. Data processing will be performed in compliance with the European Union General Data Protection Regulation (GDPR) 2016/679, and all applicable national laws to ensure data governance policies and guidelines, including data ownership, data access, data sharing, and data use agreements are managed properly.

### Sample size

The sample size for the Registry is based on the intent to characterize the safety and effectiveness data in a real-world patient population. The target is to enroll up to 3000 patients at up to 200 sites globally and follow them up to 5 years after enrollment. This sample size should provide a high degree of statistical precision on home and office blood pressure changes and a high probability of observing rare safety events; with a sample size of 3000 subjects, there is a 95% probability of observing events that occur at a population rate as low as 0.1%.

### Statistical analysis

Every effort will be made to minimize the amount of missing data. Statistical analyses will be descriptive. Continuous variables will be summarized as mean ± standard deviations and median (range) with interquartile range. Categorical variables will be summarized as frequencies and percentages. Analysis of clinical effectiveness and safety (Table 2) data will be performed on the total study population and in clinically relevant participant subgroups (e.g., based on age, history of chronic kidney disease, heart failure, atrial fibrillation, sleep apnea, diabetes mellitus, etc.). An additional analysis will determine whether BP parameters can help predict patients that may be more likely to respond to uRDN. Subgroup analyses and multivariable analyses will be performed.

**Table 2:** Standard of care parameters collected, effectiveness and safety assessments

Standard of care parameters
Demographics
Medical history
Office blood pressure
Medication usage
Short Form-12 quality-of-life questionnaire
Socioeconomic and healthcare resource use parameters
24-h ambulatory blood pressure monitoring
Electrocardiogram
Cardiac imaging
Urine chemistry
Blood chemistry
Renal artery imaging
Renal denervation procedure
Cardiovascular procedures and treatments

Effectiveness assessments
Reduction in average home systolic/diastolic BP in mmHg compared with enrollment
Reduction in average office systolic/diastolic BP in mmHg compared with enrollment
Reduction in average ambulatory systolic/diastolic BP in mmHg (daytime, nighttime and 24-h) compared with enrollment
Proportion of patients with home, office and ambulatory systolic BP reductions of ≥ 5 mmHg, ≥ 10 mmHg, and ≥ 15 mmHg post procedure
Proportion of patients meeting BP control criteria at follow-up (according to current guidelines)
Change in office/home/ambulatory pulse pressure in mmHg at follow-up compared with enrollment
Change in office/home/ambulatory heart rate in beats/min at follow-up compared with enrollment
Proportion of subjects without any antihypertensive treatment at follow-up, with analysis stratified by number of antihypertensive medications at enrollment
Analysis of quarterly home BP in mmHg at follow-up
Change in Short Form-12 quality-of-life scores during follow-up compared with enrollment

Safety assessments
All-cause mortality
Hospitalization for major cardiovascular- or hemodynamic-related events (e.g., heart failure, atrial fibrillation)
Hypertensive crisis or symptomatic hypotension
New-onset stroke, transient ischemic attack, cerebrovascular accident
Acute myocardial infraction
Any coronary revascularization
Major vascular complication (e.g., clinically significant groin hematoma, arteriovenous fistula, pseudoaneurysm) requiring surgical repair, interventional procedure, thrombin injection, or blood transfusion)
New renal artery stenosis > 70% confirmed by CTA/MRA
Renal artery perforation or dissection requiring an invasive intervention
New-onset (acute) end-stage renal disease (eGFR < 15 mL/min/m^2^ or need for renal replacement therapy)
Significant decline in renal function, defined as a ≥ 50% increase in serum creatinine (mg/mL)

BP, blood pressure; CTA, computed tomography angiography; eGFR, estimated glomerular filtration rate; MRA, magnetic resonance angiography

## Discussion

Hypertension remains the most prevalent cardiovascular risk factor and lowering BP in hypertensive patients reduces the risk of major cardiovascular events [22].

The RADIANCE-HTN SOLO study, in which untreated patients with hypertension were included, demonstrated the BP-lowering effect of uRDN at 2 months; this BP-lowering effect was maintained at 36 months when uRDN patients needed fewer antihypertensive medications than sham patients [23]. The RADIANCE-HTN TRIO trial confirmed the ability of uRDN to lower BP also in patients with resistant hypertension. In addition, observational data from the European multicenter ACHIEVE study confirmed the benefits of uRDN in patients with resistant hypertension [16]. Over 12 months of follow-up, decreases from baseline in mean office and 24-h ambulatory systolic BP were 15 mmHg and 7.5 mmHg, respectively (both *p* < 0.001) [16]. The SPYRAL HTN-OFF MED pivotal trial, in which patients with uncontrolled BP were treated with radiofrequency RDN demonstrated significant reductions in BP in absence of AHM at 3 months. Interestingly, the rate of hypertensive urgencies following RDN was significantly less (9.6%) as compared to sham (17%) between baseline and 3 months [24].

Besides randomized, sham-controlled trials, large-scale registries are particularly relevant for detection of rare events and for collecting information when devices are used in real-world conditions treated across multiple geographies outside of the confines of rigorously conducted clinical trials which increase their internal validity but with strict inclusion/exclusion criteria that lower their external validity or applicability [25]. These registries represent an important source of clinical data to support the long-term safety and effectiveness claims in a broader population, which will include subgroups under-represented in the randomized controlled trials to date. Indeed, the Global Symplicity Registry investigating radiofrequency RDN has provided interesting insights into the BP-lowering efficacy of the procedure out to 3 years [26], the response of patient subgroups with high and very high cardiovascular risk [27] as well as those with and without CKD and the potential impact of the procedure on time in therapeutic range and the subsequent consequences on cardiovascular events [27]. Long-term follow-up after radiofrequency renal denervation has also shown the BP-lowering effect of RDN maintained out to 5 years post procedure in the absence of escalating antihypertensive medications over time [28].

Ambulatory, home, and office BP measurements provide complementary information [25]. While ambulatory and office BP measurements were used in most of the randomized clinical trials and registries, limited information on the impact of RDN on home BP at long-term follow-up is currently available. The GPS Registry will provide the unique opportunity to assess teletransmitted home BP every 3 months with a dedicated telemetric device. Out-of-office home BP measurements are more reproducible than office measurements and are more closely associated with hypertension mediated organ damage (HMOD) and the risk of cardiovascular events and are thus recommended by several hypertension management guidelines [6, 8, 20]. Furthermore, the use of HBPM will facilitate increased data collection in patients over time to see how their BP evolves following uRDN. It will also permit ongoing data collection in the GPS Registry even in the event of further outbreaks of the COVID-19 or other pandemic [29]. As has been shown, one of the biggest limitations of a registry is losing subjects to follow-up. A benefit of collecting home BP is that the data will be automatically uploaded to the cloud, which will enable better long-term follow-up of patients regardless of attendance for office visits and it will allow for the collection of BP between the visits.

## Conclusion

The GPS Registry will collect relevant information on the use of uRDN under real-world conditions to evaluate the safety and effectiveness of this procedure in treating uncontrolled hypertension in a large cohort of patients.

## Data Availability

The datasets will not be publicly available because patient consent in each institute does not allow for such data publication. The corresponding author will respond to inquiries on data analyses.
